# High and low-risk specialties experience with the U.S. medical malpractice system

**DOI:** 10.1186/1472-6963-13-465

**Published:** 2013-11-06

**Authors:** Aaron E Carroll, Jennifer L Buddenbaum

**Affiliations:** 1 Center for Health Policy and Professionalism Research, Indiana University School of Medicine, Indianapolis, IN, USA; 2 Center for Pediatric and Adolescent Comparative Effectiveness Research, Indiana University School of Medicine, Indianapolis, IN, USA; 3 HITS 4099C, 410 West 10th St, Indianapolis, IN 46202, USA

## Abstract

**Background:**

“High-liability risk specialties” tend to be the focus of medical malpractice system research and debate, but concerns and fears are not limited to this group. The objective of this study was to examine whether “high-liability risk” medical specialties have a different experience with the malpractice system than “low-liability risk” specialties.

**Methods:**

We reviewed claims data from the Physician Insurers Association of America’s Data Sharing Project between January 1985 and December 2008. We used linear regression, controlling for year, to determine how liability risk affected outcomes of interest.

**Results:**

In high-liability risk specialties, 33% of claims result in indemnity payments compared to 28% for low-liability risk specialties (p < 0.001). The average indemnity payment for high-liability risk specialties was $315,314 compared to $267,146 for low-liability risk specialties (p = 0.25). Although only a small percentage of claims go to trial, low-liability risk specialties have significantly more claims that are ultimately dropped, withdrawn or dismissed, while high-liability risk specialties have significantly more claims that result in plaintiff settlement (p < 0.001).

**Conclusions:**

Malpractice risk exists for all specialties. Variability in indemnity costs are found in both high- and low-liability risk specialties. Differences in the reasons for which claims are initiated for high- and low-liability risk specialties likely necessitate different risk management solutions.

## Background

“High-liability risk specialty” or “High-risk specialty” are terms commonly utilized within the medical professional liability (MPL) system (i.e., medical malpractice system) research literature [1-10]. These terms usually refer to those medical specialties that are assumed to be at higher risk for malpractice suits, and therefore are often perceived to be the most affected by high or rising liability costs. Exactly which medical specialties are defined as “high-risk” varies, although, in general, they tend to be more procedure oriented. For example, in Studdert and colleagues’ study of defensive medicine among high-risk specialist physicians, key-informants identified six specialties as being high-risk—emergency medicine, general surgery, neurosurgery, obstetrics/gynecology, orthopedic surgery, and radiology [8]. In Kessler and colleagues’ study on the impact of malpractice reform and physician supply, high-risk specialties were identified based on malpractice premiums [9]. In this case five specialties were identified – obstetrics/gynecology, surgery, anesthesiology, emergency medicine, and radiology.

While these high-risk specialties continue to be the focus of research and debate, concerns and fears are not limited to these groups of providers. A 2009 national survey of physicians found that physicians in typically lower liability-risk specialties, such as primary care, expressed as much concern about malpractice as physicians in high-risk specialties [11]. Another national survey of physicians done in 2008 also found that concerns about malpractice liability were pervasive, with 67.7 percent of physicians expressing agreement or strong agreement [12]. However, this survey did find that those specialties generally defined as “high-risk” such as emergency physicians, obstetricians-gynecologist, and surgical specialists, did express greater concern than those specialties traditionally viewed as lower risk such as adult primary care physicians and pediatricians. Regardless of the difference, a significant number of physicians in all specialties fear malpractice lawsuits, and may therefore feel pressure to practice defense medicine to avoid them [8].

However, much of this is supposition. Some have speculated that this pervasive fear may stem from physicians lacking access to accurate and objective data about their risk of being sued, and how this risk differs from peers in other specialties [12]. Another speculation is that physicians are subject to the well-documented human tendency to overestimate the risk of rare events [12]. Do physicians in high-liability risk specialties really have that vastly different of experience with the MPL system than their low-liability risk peers? Or, instead, does the universal concern expressed across all physician specialties point to a more analogous experience?

The primary aim of this study is to look at whether those medical specialties traditionally labeled as having high-liability risk have a different experience with the MPL system than their peers in those specialties traditionally thought of as having lower-liability risk. Specifically we looked at data regarding adjudication status of claims, indemnity payment amounts, and claim related expenses. The purpose of this analysis was not to analyze how future reform might change the malpractice system, or the health care system in general. Instead we wished to provide a clearer description of what has occurred historically in the MPL system, and to shed light on whether the distinction between high-liability risk and low-liability risk specialties is truly warranted in terms of research and debate regarding the MPL system.

## Methods

### Selection of data source

To our knowledge, there are three primary sources of data available on medical malpractice judgments and awards that include data from the entire population of US physicians: 1) National Practitioner Data Bank (NPDB) [13]; 2) Jury Verdict Research (JVR) [14]; and 3) Physician Insurers Association of America Data Sharing Project (PIAA DSP) [15]. Each of these data sources has its advantages and disadvantages. The NPDB is probably the most comprehensive source of information about claims, however it is not possible to query the data based on a specific physician specialty. Therefore this data source could not be used for our analysis. The JVR collects data on jury verdicts only as reported to it by plaintiff’s attorneys, court clerks, and stringers. Since jury verdicts represent a very small percentage of medical malpractice claims, and these verdicts are significantly higher than the average settlement, this data provides a skewed and limited picture of the medical malpractice system. Another option available to researchers interested in this topic is to secure data from a single insurer. While this type of option may allow access to individual physician level data, the sample will represent an even smaller fraction of the population of medical providers from across the United States than the PIAA sample.

Therefore for this study we reviewed medical malpractice claims reported to the PIAA DSP. PIAA is a national trade organization which currently has a membership of 60 domestic MPL companies that are owned and/or operated by doctors, providers and hospitals. These member companies insure 325,000 medical practitioners in the United States [15]. Since 1985, over half of these 60 companies have voluntarily participated in the PIAA DSP, and this represents about 25% of all medical malpractice claims in the United States at a given time. While it is true that this database is not universally comprehensive, none are. Moreover, it contains information not available in the other two national databases, such as information on claims that are not ultimately paid, and specialty of the defendant. And, in comparison to individual insurer’s databases, the PIAA database provides a more generalizable set of data in that it represents medical malpractice claims from across the United States.

#### PIAA DSP

The primary goal of the PIAA DSP is to provide data to member companies to inform risk management programs that are aimed at reducing the incidence of patient injury and thereby reducing physician exposure to MPL claims. The data for the PIAA DSP are collected in a generic, anonymous format on a biannual basis. Data is available within the PIAA DSP for 28 different specialties. Data collected includes information about the claimant, the insured, payments, expenses, and claim description and causation. Diagnostic information and procedures are submitted using both International Classification of Diseases, 9^th^ revision, Clinical Modification (ICD-9) codes and PIAA-designated procedure codes [16]. Personnel at the member insurance companies who have experience handling medical malpractice claims within the claims and risk management departments are responsible for coding the data which is submitted to the DSP. These individuals have various professional backgrounds including medicine, nursing, and law and many have undergone ICD-9 coding training and certification. PIAA also provides its own training for these coders.

### Data variable definitions

• Allocated loss adjustment expenses (ALAE): All expenses paid in the process of administering or adjudicating a claim including investigative, attorney fees, expert witness fees, court costs, securing of medical records, etc [17].

• Adjudication status: How claims are resolved, including settlement, involuntary dismissal, jury verdict, alternative dispute resolution (ADR) (binding arbitration or non-binding arbitration), or contract liability agreement [17].

• Indemnity payout: Settlements or awards made directly to plaintiffs as a result of claim resolution process [17]. The indemnity payout amount does not include ALAE.

• Medical misadventure: A descriptive terminology relating to an alleged departure from accepted medical practice [17]. There are 19 types of medical misadventures that are coded within the PIAA DSP.

• “High-liability risk” and “low-liability risk” specialties: There is no one common definition for these terms. Therefore we defined these specialty groupings based on distinctions that were common across previous studies of the MPL system [8,9,11]. For our purposes, we defined emergency medicine, general surgery, obstetrics and gynecologic surgery, and radiology as our “high-liability risk” specialties, while general and family practice, internal medicine, and pediatrics were defined as our “low-liability risk” specialties.

### Data analysis

We worked in conjunction with PIAA to perform a query of its DSP database focusing on claims reported to the system as of April 2010 that had a closing date between January 1, 1985 and December 31, 2008. Only those data about closed claims were included in the analysis. Closed claims are defined as those claims where matters have been definitely resolved, regardless of whether an indemnity payout to the plaintiff occurred. Additionally the data pull was limited to the following medical specialties: emergency medicine, general surgery, obstetrics and gynecologic surgery, and radiology (high-risk), as well as general and family practice, internal medicine, and pediatrics (low-risk). All monetary amounts presented in the Results section are represented in 2008 dollar values.

We used linear regression, controlling for year to adjust for secular trends, to determine how high-malpractice-risk and primary care specialties affected outcomes of interest. We performed all calculations using the STATA 9.0 statistical package (STATA Corporation, College Station, Texas).

## Results

Between January 1, 1985 and December 31, 2008, 142,336 closed claims were reported to the PIAA DSP database for the specialties of emergency medicine, general surgery, obstetrics and gynecologic surgery, and radiology (high-liability risk) (N = 75,252), as well as general and family practice, internal medicine, and pediatrics (low-liability risk) (N = 67,084).

### Payouts and expenses

**Figure 1:**
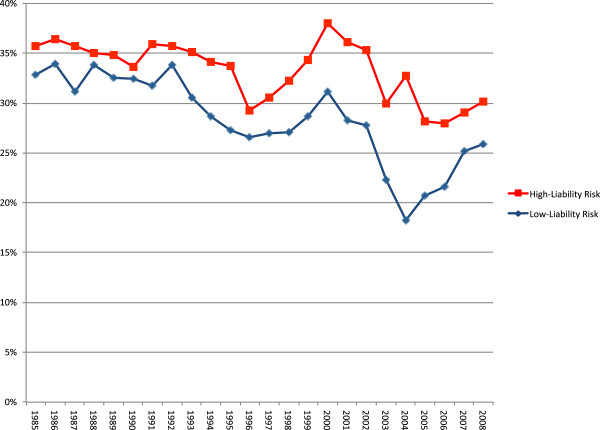
Indemnity payout rate, 1985–2008.

For the high-liability risk specialties, 33% of claims resulted in an indemnity payout, compared to 28% for the low-liability risk specialties (Table 1). Figure 1 compares the indemnity payout rate by year for the high and low-liability risk specialties. After controlling for the year of the claim, a statistically significant difference (p < 0.001) was seen in the indemnity payout rate between the two risk groupings. Internal medicine had the lowest indemnity payout rate (25%) while obstetric and gynecologic surgery had the highest (35%).

**Table 1:** All claim types, 1985-2008

	**Indemnity payout rate**	**Total indemnity**	**% of Total indemnity payout**	**Average indemnity**	**Average ALAE**
**High-liability risk specialties**	**33%**	**$7,897,041,786**	**61%**	**$315,314**	**$28,836**
Emergency medicine	26%	$283,465,068	2%	$252,868	$27,385
General surgery	34%	$2,160,833,430	17%	$254,305	$27,108
Obstetric and gynecologic surgery	35%	$4,420,235,589	34%	$385,777	$32,973
Radiology	29%	$1,032,507,700	8%	$260,143	$22,524
**Low-liability risk specialties**	**28%**	**$5,056,803,021**	**39%**	**$267,146**	**$26,581**
General and family practice	32%	$1,984,068,494	15%	$225,847	$23,550
Internal medicine	25%	$2,340,353,537	18%	$285,479	$28,106
Pediatrics	28%	$732,380,991	6%	$376,352	$31,397

**Figure 2:**
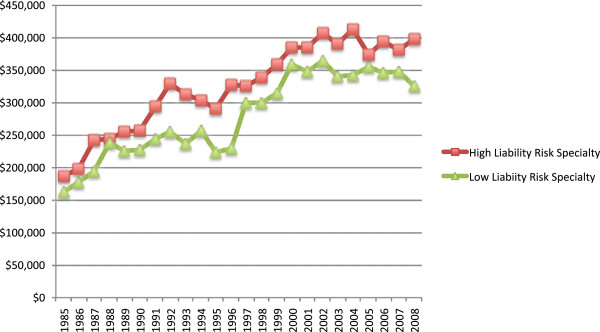
Average indemnity payout, 1985–2008.

As a percentage of total indemnity payouts, high-liability risk specialty indemnity payouts accounted for 61% compared to 39% for low-liability risk specialties (Table 1). Obstetric and gynecologic surgery accounted for the highest percentage of total indemnity payouts by far (34%) followed by internal medicine (18%), general surgery (17%), and general and family practice (15%). The average indemnity payment for high-liability risk specialties was $315,314 compared to $267,146 for the low-liability risk specialties (Table 1). However, this difference was not statistically significant (p = 0.25). Figure 2 shows the average indemnity payout over time. Interestingly, of the seven specialties included in this study, pediatrics and internal medicine had the second and third highest average indemnity payment respectively. Only obstetrics and gynecologic surgery had a higher average indemnity payment at $385,777. Median indemnity payouts in 2008 ranged from a low of $175,000 for internal medicine to a high of $250,172 for pediatrics.

The average ALAE was slightly higher for the high-liability risk specialties than for the low-liability risk specialties (Table 1). However this difference was also not statistically significant (p = 0.23). Within the PIAA DSP database, ALAE can be further sub-categorized into defense attorney expense, expert witness expense, and other expense. The breakdown of ALAE across these categories is similar for the two risk categories with defense attorney expenses accounting for the largest proportion of ALAE at 74% for high-liability risk specialties and 72% for low-liability risk specialties. The remaining expenses are divided evenly between expert witness and other expenses.

### Adjudication status

**Figure 3:**
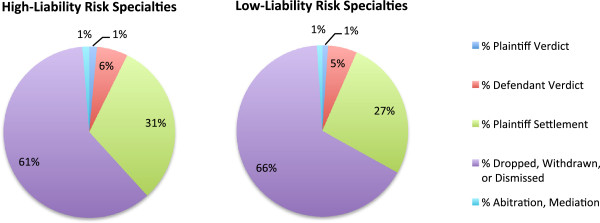
Claims by adjudication status, 1985–2008.

For both groups only a small percentage of claims go to trial (7% high-liability risk vs. 6% low-liability risk). Differences were seen in the percentage of claims that result in a plaintiff settlement, and in the percentage of claims that are dropped withdrawn, or dismissed (Figure 3). The low-liability risk specialties have significantly more claims that are ultimately dropped, withdrawn or dismissed (60% vs. 66%) while the high-liability risk specialties have significantly more claims that result in a settlement for the plaintiff (31% vs. 26%). Even after controlling for the year of the claims, to compensate for secular trends, both of these results demonstrated a statistically significant difference (p < 0.001).

Table 2 shows data for average indemnity payments broken out by final adjudication status of the claims. Indemnity payments were higher for the high-liability risk specialties if the claim resulted in a plaintiff settlement or was resolved by alternative dispute resolution (ADR)/mediation. However, the differences in average indemnity payment were not statistically significant (p = 0.33 and p = 0.18 respectively). Low-liability risk specialties on the other hand had a significantly higher average indemnity payment when the claim went to trial and resulted in a plaintiff verdict (p <0.05). Pediatrics appears to be the primary driver of this difference.

**Table 2:** Indemnity payout by adjudication status, 1985-2008

	**Average indemnity payment**				
	**Plaintiff verdict**	**Defendant verdict**	**Plaintiff settlement**	**Dropped, withdrawn or dismissed**	**Arbitration, mediation**
**High-liability risk specialties**	**$504,318**	**NA**	**$306,096**	**NA**	**$317,741**
Emergency medicine	$336,857	NA	$245,925	NA	$235,693
General surgery	$461,492	NA	$242,864	NA	$298,619
Obstetric & Gynecologic Surgery	$576,843	NA	$376,800	NA	$358,620
Radiology	$377,890	NA	$255,952	NA	$279,365
**Low-liability risk specialties**	**$518,733**	**NA**	**$256,513**	**NA**	**$267,838**
General and family practice	$421,712	NA	$216,933	NA	$272,569
Internal medicine	$565,375	NA	$273,044	NA	$259,368
Pediatrics	$676,754	NA	$367,005	NA	$276,459

No significant difference was seen between the high-liability risk and low-liability risk specialties concerning average ALAE for claims that go to trial (Table 3). However, after controlling for year, ALAE for defending claims that resulted in a plaintiff settlement (p < 0.001), that were dropped, withdrawn or dismissed (p <0.05), and that were settled via arbitration or mediation (p <0.01) were significantly different between groups.

**Table 3:** ALAE by adjudication status, 1985-2008

	**Average ALAE**				
	**Plaintiff verdict**	**Defendant verdict**	**Plaintiff settlement**	**Dropped, withdrawn or dismissed**	**Arbitration, mediation**
**High-liability risk specialties**	**$104,469**	**$86,089**	**$39,244**	**$15,482**	**$62,856**
Emergency medicine	$95,358	$101,135	$39,788	$14,424	$53,523
General surgery	$104,036	$79,709	$33,683	$15,087	$56,076
Obstetric & Gynecologic surgery	$108,135	$91,419	$45,538	$16,493	$73,131
Radiology	$92,085	$76,162	$33,117	$14,340	$50,166
**Low-liability risk specialties**	**$104,451**	**$80,165**	**$39,059**	**$15,297**	**$68,472**
General and family practice	$104,469	$71,954	$33,789	$12,718	$61,580
Internal medicine	$106,088	$83,670	$41,693	$16,900	$71,865
Pediatrics	$97,159	$92,793	$51,940	$17,173	$87,357

### Medical misadventures

A difference between the high-liability and low-liability risk specialties was also observed when comparing the types of medical misadventures coded as the primary issue of concern for closed claims between 1985 and 2008. *Improper performance* (31%) was the most prevalent medical misadventure code for high-liability risk specialty claims. *Improper performance* refers to those claims where an act performed by a physician has resulted in a procedure being performed incorrectly [18]. The next two most frequently coded medical misadventures were *errors in diagnosis* (23%) and *no medical misadventure* (21%). *No medical misadventure* refers to claims brought against a physician who had little or no contact with the patient during the event in question or in cases where other medical or legal concerns (e.g., equipment malfunctions, breach of confidentiality) are the primary issue behind the claim [16].

*Errors in diagnosis* (31%) was the most prevalent medical misadventure code for low-liability risk specialty claims. *No medical misadventure (23%)* and *improper performance* were the next most frequently coded medical misadventures for this group of physicians.

## Discussion

Our data demonstrate that high-liability risk and low-liability risk specialties do have different experiences with the MPL system. In general, high-liability risk specialties have more claims overall, as well as more claims that result in an indemnity payment. Overall indemnity payments in the high-liability risk specialties, therefore, are much higher than those from the low-risk specialties. The reasons attributed to claims are also different, as are the mechanisms by which those reasons arise. High-liability risk specialties see more claims due to improper performance of a procedure, while low-liability risk specialties have more claims where the primary issue of concern is diagnostic error. This finding is of interest and warrants further exploration in future work. Such research might want to focus on the differences among specialties in devising approaches to limit claims. “One-size-fits-all” interventions may fail to impact low risk specialties and high-risk specialties alike.

There are also similarities, however, that show in many ways, there is a “common” malpractice experience shared across all specialties regardless of whether they are categorized as high or low risk. All specialties had a significant number of claims, meaning that the risk of lawsuits, and the likely fear of being sued, exists across the specialty spectrum. The majority of claims for both high and low-liability risk specialties also get dropped, withdrawn, or dismissed. And, in both groups, only a small percentage of cases ever go to trial with an even smaller percentage resulting in a favorable verdict find for the plaintiff. Research to date has focused on the monetary costs of indemnity payments and defensive medicine. It may be, however, that the significant cost to physicians themselves has been relatively overlooked. After all, claims have a significant impact on physicians in terms of time, stress, added work, and damaged reputation. These costs occur regardless of the fact that few cases cost actual money in terms of indemnities.

Other recent work in this area supports many of our findings. Jena and colleagues [10] published a study in 2011 looking at malpractice risk according to physician specialty. This same group has also published work on how defense costs [19] and litigation outcomes [20] vary according to physician specialty. These studies utilized data from a large, physician-owned professional liability insurer that provided coverage to physicians in every state. The use of this data set allowed for analysis at the physician level, which our PIAA DSP data did not and therefore these studies were able to provide a more indepth description of risk at the individual specialty level. However, the PIAA DSP dataset utilized in our study does offer certain advantages over the individual insurer dataset. The PIAA DSP includes a significantly larger sample of claims to analyze from across the United States. Between 2002 and 2005 the PIAA DSP had 38,173 closed claims, compared to 10,056 claims for the individual insurer dataset. Our study also contains more recent data, including claims closed through 2008. The other studies cited here include claims closed up to 2005 at best.

As with all research, there are limitations to this work that warrant consideration. The PIAA DSP is not fully comprehensive, covering about 25% of United States medical malpractice claims. However, it is the only large repository of malpractice data that can be analyzed by specialty. Moreover, these data are the sole option for studying claims with no indemnity payment, which are the majority of overall claims. We also lack data on the number of physicians covered in each specialty, which is why we focus on comparisons and not on overall rates of claims per specialties. Finally, we present no data on the costs of malpractice insurance to physicians or the health care system. While important to policy decisions that was not the objective of this study.

## Conclusions

Much work on malpractice has focused on the high-liability risk specialties. In this study, we attempted to provide a comparison between those specialties traditionally viewed as high-liability risk and those specialties generally felt to be at low risk, so as to judge if significant differences exist. The differences by which cases arise and reasons behind claims being initiated points to the need for different risk management solutions in order to minimize the risk of claims for these two groups. While systems all over the country have focused on reducing exposure to malpractice claims, interventions have less commonly acknowledged that malpractice claims experience differs among specialties. Significant indemnity costs are found in both high and low-liability risk specialties, however, suggesting that even low risk specialties should be considered when addressing indemnity payments instead of aggregating specialties by perceived risk. None of this negates the fact that malpractice risk exists for all specialties, however, and remains something that is concerning for physicians. Reforms aimed to address these issues should focus on the causes and rationale for these fears, perhaps, and less on the claims themselves.

## Competing interests

The authors have no competing interests to declare related to this work.

## Authors’ contributions

AC and JB conceived the study, and participated in its design and coordination. Both authors also performed statistical analysis and helped to draft the manuscript. Both authors read and approved the final manuscript.

## Pre-publication history

The pre-publication history for this paper can be accessed here:

http://www.biomedcentral.com/1472-6963/13/465/prepub
